# Understanding continuance intention toward AI-driven healthcare services: the moderating role of e-Health Literacy

**DOI:** 10.1038/s41598-026-53246-4

**Published:** 2026-05-14

**Authors:** Yuan Jiang, Shijie Wu, Minghao Kong

**Affiliations:** Shanghai Tenth People’s Hospital, School of Medicine, Tongji University, Shanghai, China

**Keywords:** AI-driven healthcare, Continuance intention, E-Health Literacy, Privacy risk, PLS-SEM, UTAUT, Digital health, Information systems and information technology, Science, technology and society

## Abstract

AI-driven healthcare services are increasingly used to support health consultation, symptom triage, appointment navigation, and routine health management. However, sustaining users’ post-adoption engagement remains challenging, particularly because users differ in their ability to access, evaluate, and apply online health information. This study examines the determinants of continuance intention toward AI-driven healthcare services and investigates whether e-Health Literacy moderates selected relationships in the post-adoption process. Drawing on the Unified Theory of Acceptance and Use of Technology, the Information Adoption Model, and Privacy Calculus Theory, this study developed an integrated model of continuance intention. A cross-sectional survey was conducted among 580 users of AI-driven healthcare services in Shanghai, China. Data were analyzed using Partial Least Squares Structural Equation Modeling. Source trustworthiness, information quality, and effort expectancy were positively associated with performance expectancy. Performance expectancy, effort expectancy, and perceived interactivity were positively associated with continuance intention, whereas perceived privacy risk was negatively associated with continuance intention. e-Health Literacy positively moderated the relationship between information quality and performance expectancy and negatively moderated the relationship between effort expectancy and continuance intention. However, the moderation effects were small in magnitude and should be interpreted cautiously. This study extends post-adoption research on AI-driven healthcare services by showing how users’ e-Health Literacy may shape the relative importance of information quality and ease of use. The findings suggest that inclusive AI healthcare design should combine trustworthy, high-quality information with interfaces that reduce use barriers for users with lower e-Health Literacy.

## Introduction

Artificial intelligence (AI) is increasingly embedded in digital healthcare services, reshaping how individuals access health information, navigate medical resources, and manage routine health needs. AI-driven healthcare services now include intelligent triage systems, symptom-checking tools, personalized health information recommendation, AI-assisted appointment support, and conversational health consultation functions. More recently, the emergence of large language models and generative AI has further expanded the possibilities of natural language interaction in healthcare, while also raising concerns about information accuracy, accountability, privacy, and user trust^1–4^. In large urban health systems such as Shanghai, these services are increasingly viewed as part of the broader digital health infrastructure that may help improve service accessibility and reduce pressure on traditional healthcare institutions. Furthermore, the diffusion of generative AI innovations and selective knowledge dynamics across diverse user groups plays a pivotal role in shaping organizational and individual healthcare outcomes^5,6^.

China provides an important context for examining users’ sustained engagement with AI-driven healthcare services. The diffusion of online medical services has accelerated alongside the rapid development of mobile internet, hospital-based digital platforms, and internet hospitals. According to the 54th Statistical Report on China’s Internet Development, the number of online medical users in China reached 365 million by June 2024, accounting for 33.2% of all internet users (China Internet Network Information Center^7^. This expansion indicates growing public exposure to digital health services. However, rapid adoption does not necessarily lead to sustained use. Many users may try AI-driven or internet-based healthcare services for occasional consultation, appointment booking, or symptom inquiry, but may not incorporate these services into their long-term health management practices. Therefore, understanding continuance intention is essential for evaluating whether AI-driven healthcare services can generate sustainable value beyond initial trial use.

Continuance intention differs from initial technology acceptance because it is shaped by users’ accumulated post-adoption experiences. In the post-adoption stage, users have already formed direct perceptions of whether a digital service is useful, easy to operate, trustworthy, and safe. Bhattacherjee’s expectation-confirmation logic suggests that continued use depends less on novelty and more on whether the service continues to meet users’ expectations and perceived needs^8^. This distinction is particularly important in AI-driven healthcare. Unlike general digital services, AI-driven healthcare services involve health-related decision support, sensitive personal information, and varying degrees of algorithmic opacity. Users’ willingness to continue using such services may therefore depend not only on convenience and perceived usefulness, but also on the quality of health information, the credibility of the source, the responsiveness of interaction, and concerns about privacy risk.

Prior studies have applied technology acceptance and continuance models, such as the Unified Theory of Acceptance and Use of Technology (UTAUT), to explain users’ adoption and continued use of digital health and mobile health services^9–12^. These studies have established the importance of performance expectancy and effort expectancy in shaping behavioral intention. However, AI-driven healthcare services are not only technological tools but also information-intensive services. Users must evaluate whether AI-provided information is accurate, relevant, understandable, and trustworthy. Therefore, the Information Adoption Model (IAM) provides a useful complementary perspective by distinguishing between information quality as a central cue and source trustworthiness as a peripheral cue in information evaluation^13^. At the same time, because AI-driven healthcare services often require disclosure of symptoms, medical histories, and other sensitive data, Privacy Calculus Theory helps explain how perceived privacy risks may offset perceived benefits and reduce continuance intention^14–16^.

Despite these insights, two important gaps remain. First, existing research often gives insufficient attention to the specific informational and risk-related features of AI-driven healthcare services. In such services, perceived usefulness may depend not only on ease of use but also on whether users trust the source behind AI-generated recommendations and whether they perceive the information as reliable and relevant. This issue is especially salient because AI-generated health information can be difficult for lay users to verify, and generative AI systems may produce fluent but inaccurate or incomplete responses^2,4^. Second, prior research often treats users as relatively homogeneous, while users differ substantially in their ability to seek, understand, evaluate, and apply online health information. These differences may shape how users respond to the same AI-driven healthcare service.

e-Health Literacy is therefore central to understanding heterogeneity in post-adoption evaluation. Norman and Skinner^17^, define e-Health Literacy as the ability to seek, find, understand, and appraise health information from electronic sources and apply such knowledge to health-related problems. Neter and Brainin^18^, further argue that e-Health Literacy extends the digital divide into the realm of online health information. In AI-driven healthcare contexts, users with higher e-Health Literacy may be better able to evaluate whether AI-generated information is accurate, relevant, and applicable to their own health needs. In contrast, users with lower e-Health Literacy may rely more heavily on interface simplicity and operational ease because complex digital interactions can increase cognitive burden and uncertainty. Thus, e-Health Literacy may not simply have a direct effect on continuance intention; rather, it may moderate how users translate information quality and effort expectancy into perceived usefulness and continued use.

To address these gaps, this study develops and tests an integrated model of continuance intention toward AI-driven healthcare services. The model combines UTAUT, IAM, and Privacy Calculus Theory to examine the roles of performance expectancy, effort expectancy, information quality, source trustworthiness, perceived interactivity, and perceived privacy risk. It further investigates whether e-Health Literacy moderates the relationships between information quality and performance expectancy, and between effort expectancy and continuance intention. Using survey data from 580 users of AI-driven healthcare services in Shanghai, China, this study addresses the following research questions:

RQ1: What technological, informational, and risk-related factors influence users’ continuance intention toward AI-driven healthcare services?

RQ2: How do information quality, source trustworthiness, and effort expectancy shape users’ performance expectancy?

RQ3: Does e-Health Literacy moderate the effects of information quality and effort expectancy in the post-adoption process?

This study makes three contributions. First, it extends research on AI-driven healthcare continuance by shifting attention from initial adoption to post-adoption intention. Second, it integrates UTAUT, IAM, and Privacy Calculus Theory to capture both benefit-related and risk-related determinants of continuance intention. Third, it introduces e-Health Literacy as a moderator, offering a more nuanced explanation of literacy-related heterogeneity in users’ evaluation of AI-driven healthcare services. Rather than claiming to fully explain the digital divide, this study focuses on how e-Health Literacy may shape the relative importance of information quality and ease of use in continued engagement with AI-driven healthcare.

## Literature review and hypotheses

### Theoretical framework

This study integrates UTAUT, IAM, and Privacy Calculus Theory because each theory explains a distinct component of users’ post-adoption evaluation of AI-driven healthcare services. UTAUT provides the baseline logic for technology continuance by emphasizing performance expectancy and effort expectancy as key determinants of behavioral intention. In a post-adoption context, users are more likely to continue using a service when they believe it improves their health management and when the service remains easy to use^10,11^.

IAM complements UTAUT by explaining how users form judgments about the usefulness of information-intensive systems. AI-driven healthcare services do not merely provide technical functions; they also generate, organize, and recommend health information. Therefore, users’ performance expectancy depends not only on system usability but also on their assessment of information quality and source trustworthiness. Information quality represents the central route of information evaluation, whereas source trustworthiness functions as a peripheral cue that helps users evaluate information when medical expertise or algorithmic transparency is limited^13^.

Privacy Calculus Theory further extends the framework by introducing the risk side of AI-driven healthcare use. Because these services often require users to disclose personal symptoms, medical histories, and other sensitive information, users may weigh perceived benefits against perceived privacy risks. Thus, perceived privacy risk is included as a negative predictor of continuance intention^14,15^.

**Fig. 1:**
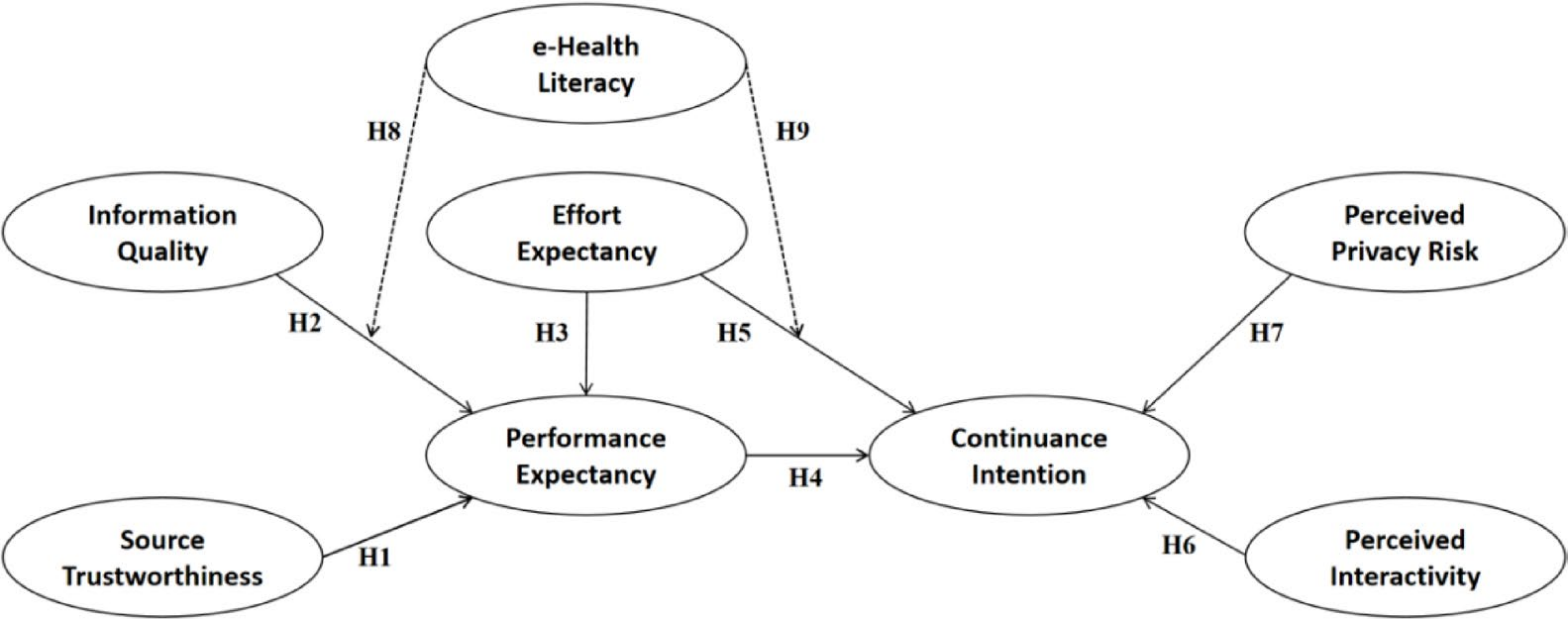
The conceptual framework.

Finally, e-Health Literacy is incorporated as a moderator because users differ in their ability to locate, understand, evaluate, and apply online health information. These differences may alter how users respond to information quality and ease of use. High-literacy users may be better able to recognize and use high-quality health information, whereas low-literacy users may be more dependent on interface simplicity. This integrated framework therefore captures both the benefit–risk evaluation and the capability-based heterogeneity underlying continuance intention toward AI-driven healthcare services. Based on this theoretical integration, the conceptual framework of this study is presented in Fig. 1.

### Antecedents of performance expectancy

Performance expectancy refers to the degree to which users believe that using a technology will help them achieve desired outcomes^10^. In AI-driven healthcare services, performance expectancy reflects users’ perception that the service can help them manage health-related tasks more effectively, obtain useful health information, and navigate medical resources more efficiently.

Source trustworthiness is particularly important in healthcare because users often lack the professional expertise needed to independently verify medical information. When AI-driven services are perceived as being supported by credible medical institutions, professional sources, or trustworthy platforms, users are more likely to perceive the service as useful. In high-risk domains such as healthcare, source trustworthiness may operate as a heuristic that reduces uncertainty and supports users’ evaluation of AI-generated information^13,19,20^. Therefore:

#### H1

Source trustworthiness positively influences performance expectancy.

Information quality refers to users’ perception that the health information provided by the service is accurate, relevant, reliable, and applicable to their needs. For AI-driven healthcare services, information quality is central because the value of the service depends heavily on whether AI-generated recommendations and health responses are perceived as useful and dependable. High-quality information can improve users’ confidence in applying the service to health management and can strengthen their perception that the service provides meaningful support^13^. Therefore:

#### H2

Information quality positively influences performance expectancy.

Effort expectancy refers to the degree to which users perceive the service as easy to learn and operate^10^. In AI-driven healthcare services, ease of use can reduce cognitive burden, especially when users must input symptoms, interpret system responses, or navigate multiple service functions. When a platform is easy to use, users can focus more on health-related tasks rather than operational difficulties. This may increase the perceived usefulness of the service (Davis, 1989^10^;. Therefore:

#### H3

Effort expectancy positively influences performance expectancy.

### Determinants of continuance intention

Continuance intention refers to users’ willingness to continue using a service after initial adoption^8^. In the post-adoption stage, users have direct experience with the service and are therefore likely to base their continued use on perceived utility, ease of operation, interaction experience, and risk assessment.

Performance expectancy is expected to be a key driver of continuance intention because users are more likely to continue using AI-driven healthcare services when they believe these services improve health management, save time, or provide useful support. Prior research on technology continuance and digital health services has consistently shown that perceived usefulness or performance expectancy plays a central role in continued use^8,9,11,12^. Therefore:

#### H4

Performance expectancy positively influences continuance intention.

Effort expectancy may also directly influence continuance intention. Even if a service is perceived as useful, users may discontinue use if it is difficult to operate or requires excessive effort. This issue is especially relevant in healthcare settings, where users may include older adults, patients with chronic conditions, and individuals with limited digital capability. A clear and easy-to-use interface can reduce barriers to repeated use and increase users’ willingness to continue using the service. Therefore:

#### H5

Effort expectancy positively influences continuance intention.

Perceived interactivity refers to users’ perception that the service provides timely, responsive, and dynamic interaction. In AI-driven healthcare services, interactive functions such as real-time responses, adaptive questioning, and conversational feedback may increase user engagement and create a sense of responsiveness. Although this study operationalizes perceived interactivity primarily through responsiveness and dynamic feedback rather than all dimensions of interactivity, these features may still encourage continued use by making the service experience more engaging and personalized (Pillai & Sivathanu, 2020). Therefore:

#### H6

Perceived interactivity positively influences continuance intention.

Perceived privacy risk refers to users’ concerns about potential loss, misuse, or unauthorized disclosure of personal information. AI-driven healthcare services often involve sensitive health data, including symptoms, medical histories, and personal identifiers. According to Privacy Calculus Theory, users weigh perceived benefits against perceived risks when deciding whether to continue using a service^15^. If users are concerned about data leakage, excessive information collection, or unclear data governance, their continuance intention may decrease^14,16,21^. Therefore:

#### H7

Perceived privacy risk negatively influences continuance intention.

### The moderating role of e-Health Literacy

e-Health Literacy refers to users’ ability to seek, find, understand, evaluate, and apply health information from electronic sources^17^. In AI-driven healthcare services, e-Health Literacy may shape how users interpret AI-generated health information and how they respond to interface complexity. Therefore, this study proposes that e-Health Literacy moderates two key relationships in the model.

First, e-Health Literacy may strengthen the effect of information quality on performance expectancy. Users with higher e-Health Literacy are more capable of evaluating whether AI-generated health information is accurate, relevant, and applicable to their own health needs. They may be better able to distinguish professional advice from unreliable health information, compare AI-generated responses with prior knowledge, and translate high-quality information into actionable value. In contrast, users with lower e-Health Literacy may have difficulty identifying differences in information quality and may therefore derive less perceived usefulness from high-quality information. Therefore:

#### H8

e-Health Literacy positively moderates the relationship between information quality and performance expectancy, such that the relationship is stronger among users with higher e-Health Literacy.

Second, e-Health Literacy may weaken the effect of effort expectancy on continuance intention. Users with lower e-Health Literacy often face greater cognitive and operational barriers when interacting with digital health services, including difficulties in understanding interface instructions, entering health information, interpreting feedback, and navigating service functions. For these users, ease of use may directly reduce anxiety and increase willingness to continue using the service. By contrast, users with higher e-Health Literacy are generally more familiar with online health resources and digital interfaces. For them, ease of use may be regarded as a basic requirement rather than a primary source of continuance motivation. Therefore:

#### H9

e-Health Literacy negatively moderates the relationship between effort expectancy and continuance intention, such that the relationship is weaker among users with higher e-Health Literacy.

## Methodology

### Study design, participants, and data collection

A cross-sectional survey was conducted in Shanghai, China, from August to October 2025. The target population consisted of adult users who had prior experience with AI-driven healthcare services, such as intelligent triage systems, AI-assisted health consultation tools, symptom-checking services, or large-language-model-enabled health question-answering functions. In this study, AI-driven healthcare services refer to digital health services that incorporate algorithmic or intelligent functions to support health consultation, symptom analysis, personalized health information provision, or healthcare service navigation. The study was not limited exclusively to generative AI applications, although generative-AI-enabled health services were included as one emerging form of AI-driven healthcare services.

To reduce coverage bias and improve the inclusion of users with different levels of digital capability, a hybrid recruitment strategy was adopted. First, the questionnaire was distributed through Wenjuanxing, a widely used online survey platform in China. This online panel was used to reach regular internet users with prior experience of digital health services. Second, to include older adults and users who might be underrepresented in purely online surveys, assisted questionnaire completion was conducted through two community health centers in Shanghai. Trained research assistants explained the study purpose, confirmed participants’ prior use of AI-driven healthcare services, and provided assistance only with operating the survey interface when needed, without influencing participants’ answers.

A total of 712 responses were initially collected. After data screening, 132 responses were excluded, including 45 responses that failed attention-check questions, 38 responses with abnormally short completion times, 32 responses showing straight-lining or highly patterned answers, and 17 responses with logical inconsistencies. The final valid sample consisted of 580 respondents, yielding a valid response rate of 81.5%. Among the valid responses, 450 were obtained from the online panel and 130 from community health centers, accounting for 77.6% and 22.4% of the final sample, respectively. Participants received a small equivalent incentive of RMB 10, either through the online platform or in the form of a practical gift of equivalent value for community participants.

### Measures and questionnaire development

The measurement items were adapted from validated scales in prior literature. Source trustworthiness, information quality, performance expectancy, effort expectancy, and continuance intention were adapted from prior research on information adoption and technology acceptance. Perceived privacy risk was adapted from Featherman and Pavlou^22^. e-Health Literacy was measured using items adapted from the eHealth Literacy Scale^17^. All constructs were measured using a seven-point Likert scale ranging from 1 = strongly disagree to 7 = strongly agree.

Before answering the questionnaire, participants were presented with a clear definition and examples of AI-driven healthcare services, including intelligent triage robots, symptom analysis tools, AI-assisted consultation systems, and large-language-model-based health question-answering tools. This procedure was used to help respondents distinguish AI-driven healthcare services from ordinary administrative health applications, such as appointment-only platforms.

Because the original scales were developed primarily in English, a translation and back-translation procedure was conducted following Brislin’s approach^23^. Two bilingual doctoral students independently translated the original English items into Chinese. The translated version was then reviewed and reconciled by the research team. A separate bilingual expert, who had not seen the original English version, back-translated the Chinese questionnaire into English. Discrepancies between the original and back-translated versions were discussed and resolved to ensure semantic equivalence.

To further improve content validity and contextual appropriateness, the questionnaire was reviewed by three experts: two professors with expertise in information systems and digital health, and one senior clinician with experience in community healthcare services. Their feedback was used to refine wording, particularly for items involving AI-driven healthcare services and e-Health Literacy.

A pretest was conducted with 35 participants, including 20 younger users and 15 older community users. These responses were not included in the final sample. The pretest indicated that some older participants found the phrase “evaluate health resources” difficult to understand. Therefore, a brief explanatory phrase was added in the Chinese questionnaire, such as “for example, distinguishing professional medical advice from unreliable online health rumors.” These revisions improved the clarity and contextual relevance of the questionnaire.

### Control variables

Gender, age, education, and health status were included as control variables because prior digital health and technology acceptance studies suggest that demographic and health-related factors may influence users’ ability, motivation, and need to use digital health services. Age and education are particularly relevant to e-Health Literacy and digital health use, as older adults and individuals with lower educational attainment may face greater barriers in accessing and evaluating online health information. Health status was included because users with chronic conditions may have stronger needs for continuous health management and may therefore differ in their intention to continue using AI-driven healthcare services. Gender was included to control for potential differences in health information-seeking behavior and digital service use.

### Data analysis

Data were analyzed using Partial Least Squares Structural Equation Modeling with SmartPLS 4. PLS-SEM was considered appropriate because the study aimed to predict continuance intention, included multiple theoretical constructs and interaction terms, and examined moderation effects^24^; Hair et al.^25^,. The analysis followed a two-step approach. First, the measurement model was assessed using indicator loadings, Cronbach’s alpha, composite reliability, average variance extracted, and the heterotrait–monotrait ratio. Second, the structural model was evaluated using path coefficients, t values, p values, confidence intervals, coefficient of determination, effect sizes, and predictive relevance. Bootstrapping with 5,000 subsamples was used to test the significance of the direct and moderating effects.

The moderation effects of e-Health Literacy were tested using interaction terms between e-Health Literacy and information quality, and between e-Health Literacy and effort expectancy. To facilitate interpretation, simple slope plots were generated for users with higher and lower levels of e-Health Literacy.

### Bias assessment

Common method bias was addressed through both procedural and statistical remedies. Procedurally, participants were informed that their responses would remain anonymous, that there were no right or wrong answers, and that the data would be used only for research purposes. The questionnaire used clear wording, and predictor and outcome constructs were separated where possible to reduce evaluation apprehension and response consistency bias.

Statistically, Harman’s single-factor test showed that the first factor explained 26.804% of the total variance, below the commonly used 50% threshold. In addition, collinearity diagnostics were conducted using SmartPLS. As reported in Table 2, all outer VIF values were below 3.3, ranging from 1.891 to 2.740. The inner VIF values were also below 3.3, ranging from 1.008 to 1.190, suggesting that multicollinearity was not a serious concern in the structural model. Together with the procedural remedies and Harman’s single-factor test, these results suggest that common method bias was unlikely to fully account for the observed relationships. Nevertheless, because all data were self-reported and collected at one time point, common method bias cannot be completely ruled out and is acknowledged as a limitation.

Nonresponse bias was assessed by comparing early and late respondents following Armstrong and Overton’s procedure^26^. The valid responses were sorted by submission time, and the first 25% of respondents were compared with the last 25% using independent-samples t-tests. No significant differences were found across the main constructs, suggesting that nonresponse bias was unlikely to substantially influence the findings.

Because the data were collected through both an online panel and community health centers, potential mode-related differences were also examined. Independent-samples t-tests showed no statistically significant differences between the two recruitment modes across the key constructs, including continuance intention, effort expectancy, information quality, performance expectancy, perceived interactivity, perceived privacy risk, source trustworthiness, and e-Health Literacy. Therefore, the hybrid data-collection approach was unlikely to have introduced substantial mode-related differences in the focal variables.

## Results

### Demographic profile

The demographic characteristics of the respondents are presented in Table 1. Among the 580 respondents, 278 were male and 302 were female, accounting for 47.9% and 52.1% of the sample, respectively. The age distribution included 145 respondents aged 18–30 years, 186 aged 31–45 years, 134 aged 46–60 years, and 115 aged over 60 years. Respondents also varied in educational background, with 24.0% having a high school education or below, 21.0% holding an associate degree, 41.9% holding a bachelor’s degree, and 13.1% holding a master’s degree or above. In addition, 42.1% of respondents reported having chronic conditions, indicating that a substantial portion of the sample had ongoing health management needs.

**Table 1:** Demographic characteristics of participants (*N* = 580).

Measure	Category	Frequency (*N*)	Percentage (%)
Gender	Male	278	47.90%
	Female	302	52.10%
Age	18–30 years	145	25.00%
	31–45 years	186	32.10%
	46–60 years	134	23.10%
	Over 60 years	115	19.80%
Education	High school or below	139	24.00%
	Associate degree	122	21.00%
	Bachelor’s degree	243	41.90%
	Master’s degree or above	76	13.10%
Health Status	No chronic condition (Healthy)	336	57.90%
	Has chronic condition(s)	244	42.10%

### Measurement model assessment

**Fig. 2:**
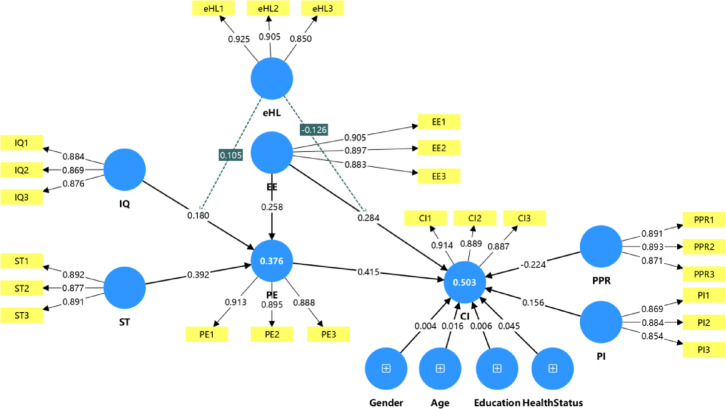
Standardized PLS-SEM results. Note Standardized path coefficients, indicator loadings, and explained variance are shown. Values inside endogenous constructs indicate R².

The measurement model was assessed by examining reliability, convergent validity, and discriminant validity. Figure 2 presents the standardized PLS-SEM results, including indicator loadings, standardized path coefficients, and explained variance. As shown in Table 2, all outer VIF values ranged from 1.891 to 2.740, below the recommended threshold of 3.3, indicating no serious indicator-level collinearity. All standardized factor loadings exceeded 0.70. Cronbach’s alpha and composite reliability values were above 0.70 for all constructs, and AVE values ranged from 0.755 to 0.808, supporting convergent validity^24^^25^;.

**Table 2:** Assessment of measurement model reliability, validity, and indicator collinearity.

Construct	Items	VIF	Factor Loading	Cronbach’s Alpha	CR	AVE
CI	CI1	2.74	0.914	0.878	0.925	0.804
	CI2	2.3	0.889			
	CI3	2.333	0.887			
EE	EE1	2.361	0.905	0.876	0.924	0.801
	EE2	2.54	0.897			
	EE3	2.284	0.883			
IQ	IQ1	2.06	0.884	0.849	0.909	0.768
	IQ2	2.062	0.869			
	IQ3	2.071	0.876			
PE	PE1	2.655	0.913	0.881	0.927	0.808
	PE2	2.429	0.895			
	PE3	2.338	0.888			
PI	PI1	1.994	0.869	0.838	0.902	0.755
	PI2	2.011	0.884			
	PI3	1.891	0.854			
PPR	PPR1	2.334	0.891	0.862	0.915	0.783
	PPR2	2.112	0.893			
	PPR3	2.182	0.871			
ST	ST1	2.263	0.892	0.864	0.917	0.786
	ST2	2.137	0.877			
	ST3	2.283	0.891			
eHL	eHL1	2.544	0.925	0.876	0.923	0.799
	eHL2	2.561	0.905			
	eHL3	2.155	0.850			

Discriminant validity was assessed using the heterotrait–monotrait ratio of correlations. As shown in Table 3, all HTMT values among the core latent constructs were below the conservative threshold of 0.85. The highest HTMT value was observed between performance expectancy and continuance intention (HTMT = 0.635), followed by source trustworthiness and performance expectancy (HTMT = 0.576), both of which remained well below the recommended cut-off^27^. These results support the discriminant validity of the measurement model.

**Table 3:** Discriminant validity assessment using HTMT.

	CI	EE	IQ	PE	PI	PPR	ST	eHL
CI								
EE	0.538							
IQ	0.347	0.087						
PE	0.635	0.389	0.41					
PI	0.35	0.219	0.253	0.203				
PPR	0.331	0.089	0.088	0.081	0.071			
ST	0.432	0.181	0.407	0.576	0.14	0.073		
eHL	0.221	0.159	0.182	0.205	0.114	0.047	0.129	

### Structural model assessment

Before assessing the structural paths, collinearity among the predictor constructs was examined. All inner VIF values ranged from 1.008 to 1.190, below the recommended threshold of 3.3, indicating no serious multicollinearity among the predictors.

**Fig. 3:**
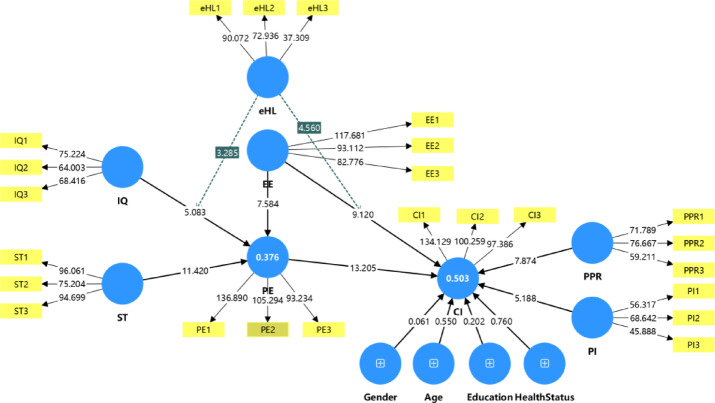
Bootstrapping results of the structural model. Note Values on paths indicate bootstrapped t statistics. Values inside endogenous constructs indicate R².

The structural model was evaluated using bootstrapping with 5,000 subsamples. The model explained 37.6% of the variance in performance expectancy and 50.2% of the variance in continuance intention, indicating moderate explanatory power. Figure 3 presents the bootstrapping results of the structural model. Values on paths indicate bootstrapped t statistics, and values inside endogenous constructs indicate R². The final path coefficients, confidence intervals, and effect sizes are reported in Table 4.

Source trustworthiness had the strongest positive association with performance expectancy (β = 0.392, t = 11.420, *p* < 0.001), supporting H1. Information quality was also positively associated with performance expectancy (β = 0.180, t = 5.083, *p* < 0.001), supporting H2. Effort expectancy showed a positive association with performance expectancy (β = 0.258, t = 7.584, *p* < 0.001), supporting H3. These findings suggest that users’ perceived usefulness of AI-driven healthcare services was shaped by both informational cues and usability-related evaluations.

Regarding continuance intention, performance expectancy was the strongest positive predictor (β = 0.415, t = 13.205, *p* < 0.001), supporting H4. Effort expectancy was also positively associated with continuance intention (β = 0.284, t = 9.120, *p* < 0.001), supporting H5. Perceived interactivity had a positive association with continuance intention (β = 0.156, t = 5.188, *p* < 0.001), supporting H6. Perceived privacy risk was negatively associated with continuance intention (β = −0.224, t = 7.874, *p* < 0.001), supporting H7. These results indicate that continuance intention was influenced by both perceived benefits and perceived risks.

The main effects of e-Health Literacy were included when estimating the interaction terms but were not treated as separate hypotheses; therefore, they are not reported as hypothesized paths in Table 4.

**Table 4:** Structural model results.

Types	Path	β	SD	T	*P*	95% CI	f²	Decision
H1	ST -> PE	0.392	0.034	11.420	*p* < 0.001	[0.323, 0.459]	0.211	Supported
H2	IQ -> PE	0.180	0.035	5.083	*p* < 0.001	[0.111, 0.248]	0.045	Supported
H3	EE -> PE	0.258	0.034	7.584	*p* < 0.001	[0.192, 0.325]	0.102	Supported
H4	PE -> CI	0.415	0.031	13.205	*p* < 0.001	[0.352, 0.476]	0.291	Supported
H5	EE -> CI	0.284	0.031	9.120	*p* < 0.001	[0.223, 0.346]	0.138	Supported
H6	PI -> CI	0.156	0.030	5.188	*p* < 0.001	[0.096, 0.215]	0.046	Supported
H7	PPR -> CI	−0.224	0.028	7.874	*p* < 0.001	[−0.280, −0.169]	0.099	Supported
H8	eHL x IQ -> PE	0.105	0.032	3.285	0.001	[0.040, 0.165]	0.018	Statistically supported; very small effect
H9	eHL x EE -> CI	−0.126	0.028	4.560	*p* < 0.001	[−0.178, −0.070]	0.033	Statistically supported; small effect
Control	Gender -> CI	0.004	0.061	0.061	0.951	[−0.114, 0.125]	0.000	Not significant
Control	Age -> CI	0.016	0.029	0.550	0.583	[−0.042, 0.073]	0.001	Not significant
Control	Education -> CI	0.006	0.031	0.202	0.840	[− 0.052, 0.067]	0.000	Not significant
Control	HealthStatus -> CI	0.045	0.059	0.760	0.447	[− 0.069, 0.163]	0.001	Not significant

Note. The main effects of e-Health Literacy were included in the moderation model but were not hypothesized separately.

Effect sizes were assessed using f². Performance expectancy had the largest effect on continuance intention (f² = 0.291), followed by source trustworthiness on performance expectancy (f² = 0.211). Effort expectancy showed small-to-moderate effects on continuance intention (f² = 0.138) and performance expectancy (f² = 0.102). Perceived privacy risk also had a small-to-moderate effect on continuance intention (f² = 0.099). Information quality and perceived interactivity showed small effects. The two interaction effects were smaller in magnitude, especially the eHL × Information Quality interaction. These results indicate that the moderation findings were statistically detectable but should not be overinterpreted as large practical effects.

### Moderation analysis

The moderation analysis provided evidence for two statistically significant moderating effects of e-Health Literacy. First, the interaction between e-Health Literacy and information quality was positively associated with performance expectancy (β = 0.105, t = 3.285, *p* = 0.001), supporting H8. This suggests that the relationship between information quality and performance expectancy was stronger among users with higher e-Health Literacy. However, the effect size was very small (f² = 0.018), slightly below the conventional 0.02 threshold for a small effect. Therefore, this moderation effect should be interpreted cautiously.

**Fig. 4:**
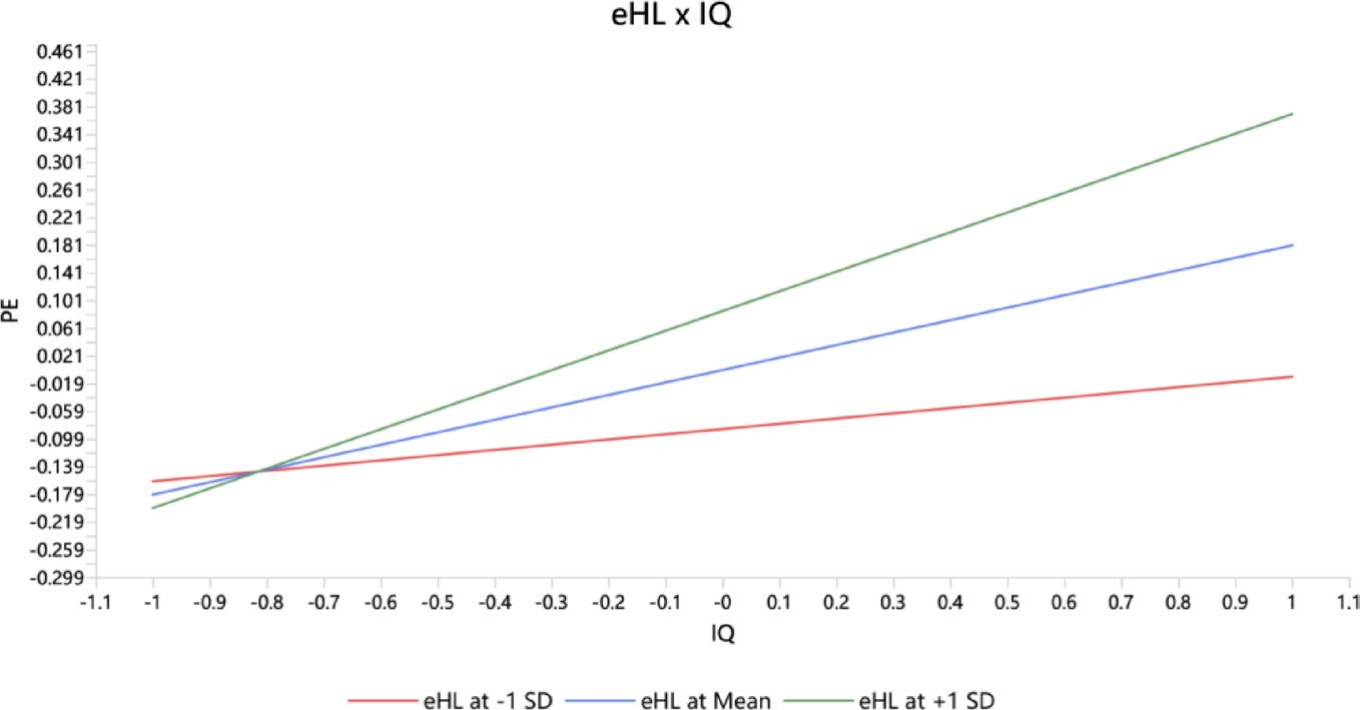
Simple slope analysis of the moderating effect of eHL x IQ.

**Fig. 5:**
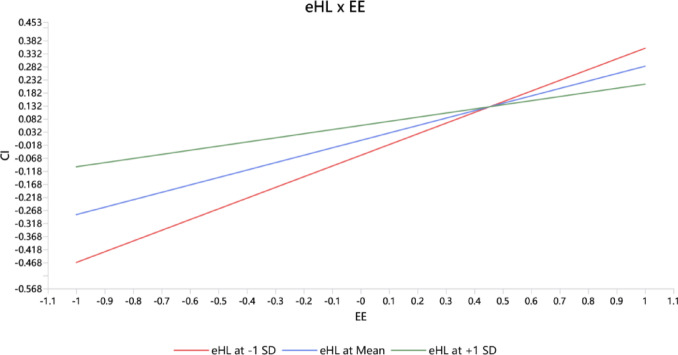
Simple slope analysis of the moderating effect of eHL x EE.

Second, the interaction between e-Health Literacy and effort expectancy was negatively associated with continuance intention (β = −0.126, t = 4.560, *p* < 0.001), supporting H9. This indicates that the positive relationship between effort expectancy and continuance intention was weaker among users with higher e-Health Literacy and stronger among users with lower e-Health Literacy. The effect size was small (f² = 0.033), suggesting a statistically significant but modest moderation effect (Figures 4, 5).

### Bias and sensitivity analysis

Nonresponse bias was assessed by comparing early and late respondents following Armstrong and Overton’s procedure. The first 25% and last 25% of valid responses were compared using independent-samples t-tests. No significant differences were found for continuance intention (t = 0.679, *p* = 0.498), effort expectancy (t = 1.759, *p* = 0.080), information quality (t = − 0.307, *p* = 0.759), performance expectancy (t = 0.287, *p* = 0.775), perceived interactivity (t = − 0.467, *p* = 0.641), perceived privacy risk (t = 0.903, *p* = 0.367), source trustworthiness (t = 0.465, *p* = 0.642), or e-Health Literacy (t = 0.920, *p* = 0.358). These findings suggest that nonresponse bias was unlikely to substantially influence the results.

Because data were collected through both an online panel and community health centers, potential mode-related differences were also examined. Independent-samples t-tests showed no statistically significant differences between the two recruitment modes across the key constructs, including continuance intention, effort expectancy, information quality, performance expectancy, perceived interactivity, perceived privacy risk, source trustworthiness, and e-Health Literacy. Therefore, the hybrid data-collection approach was unlikely to have introduced substantial mode-related differences in the focal variables.

### Predictive relevance

To further assess predictive relevance, Q²predict values were examined. As shown in Table 5, the Q²predict values for continuance intention and performance expectancy were greater than zero, indicating acceptable predictive relevance. These results suggest that the model has predictive value for the key endogenous constructs, although the results should be interpreted in conjunction with the reported effect sizes.

**Table 5:** Predictive relevance assessment.

Construct	Q²predict	RMSE	MAE
CI	0.413	0.769	0.621
PE	0.363	0.801	0.651

## Discussion

### Principal findings

This study examined the determinants of users’ continuance intention toward AI-driven healthcare services and investigated whether e-Health Literacy moderates selected relationships in the post-adoption process. By integrating UTAUT, IAM, and Privacy Calculus Theory, the study provides a more comprehensive explanation of continued use in AI-driven healthcare contexts. The findings show that performance expectancy was the strongest predictor of continuance intention, while source trustworthiness, effort expectancy, and information quality were significant antecedents of performance expectancy. Perceived privacy risk negatively influenced continuance intention, indicating that risk concerns remain important even when users perceive AI-driven healthcare services as useful.

The finding that performance expectancy strongly predicted continuance intention is consistent with UTAUT and prior continuance research^8–11^. In the post-adoption stage, users are likely to continue using a service when they perceive that it helps them manage health-related tasks, obtain useful information, and improve service efficiency. This result confirms that utility remains central to continued engagement with AI-driven healthcare services.

An important finding is that source trustworthiness had a stronger association with performance expectancy than information quality. This may appear to differ from the traditional IAM emphasis on information quality as a central-route determinant of information adoption. However, in AI-driven healthcare contexts, users often face high uncertainty, limited medical expertise, and limited transparency regarding how AI-generated recommendations are produced. Under such conditions, source trustworthiness may become a dominant heuristic for evaluating usefulness. Users may first rely on whether the service is backed by a credible hospital, medical institution, or professional source before they assess the detailed quality of the information. This finding suggests that, in high-risk and expertise-intensive contexts such as healthcare, peripheral cues may not merely supplement central-route evaluation but may serve as a prerequisite for users’ perceived usefulness of AI-generated health information.

The negative association between perceived privacy risk and continuance intention supports Privacy Calculus Theory. AI-driven healthcare services often require users to provide sensitive health information, which may intensify concerns about data leakage, excessive collection, or inappropriate secondary use. Even when users recognize the potential benefits of AI-driven services, privacy concerns may reduce their willingness to continue using them. This finding aligns with prior research on privacy concerns in digital and smart healthcare services^14,16,20,21^.

### The moderating role of e-Health Literacy

This study found two statistically significant but modest moderating effects of e-Health Literacy. First, e-Health Literacy positively moderated the relationship between information quality and performance expectancy. This suggests that users with higher e-Health Literacy may be somewhat better able to translate high-quality AI-generated health information into perceived usefulness. This pattern is consistent with a possible knowledge-amplification interpretation: users with stronger health-related digital skills may be more capable of evaluating the relevance, accuracy, and applicability of AI-provided health information. However, the effect size of this interaction was very small. Therefore, this finding should be interpreted as preliminary evidence of literacy-related heterogeneity rather than as strong evidence of a large moderation mechanism.

Second, e-Health Literacy negatively moderated the relationship between effort expectancy and continuance intention. This suggests that ease of use was more strongly associated with continuance intention among users with lower e-Health Literacy. For these users, a clear interface and easy operation may reduce cognitive burden and uncertainty. For users with higher e-Health Literacy, ease of use may be closer to a baseline expectation and therefore less decisive for continued use. This pattern is consistent with a hygiene-factor interpretation of effort expectancy, but the small effect size indicates that further research is needed to validate this explanation.

### Theoretical implications

This study makes several theoretical contributions. First, it extends research on AI-driven healthcare services by focusing on continuance intention rather than initial adoption. While prior studies have examined technology acceptance in digital health contexts, less is known about why users continue using AI-driven healthcare services after initial exposure. By emphasizing post-adoption evaluation, this study highlights the importance of performance expectancy, privacy risk, and user capability in sustained engagement.

Second, this study integrates UTAUT, IAM, and Privacy Calculus Theory to explain continuance intention in an information-intensive and risk-sensitive context. UTAUT explains the role of performance expectancy and effort expectancy, IAM explains how information quality and source trustworthiness shape perceived usefulness, and Privacy Calculus Theory explains why perceived privacy risk may reduce continued use. This integrated framework is particularly suitable for AI-driven healthcare services, where technical usability, information evaluation, and privacy concerns are closely intertwined.

Third, this study contributes to the literature on e-Health Literacy by showing that literacy-related differences may operate through moderation rather than only through direct effects. The findings suggest that e-Health Literacy may shape how users evaluate information quality and ease of use. This provides a more nuanced understanding of user heterogeneity in AI-driven healthcare services. However, because the moderation effect sizes were small, these findings should be interpreted as modest evidence of literacy-related heterogeneity rather than as definitive evidence of a large digital divide mechanism.

### Practical implications

The findings also have practical implications for the design and governance of AI-driven healthcare services. First, source trustworthiness should be prioritized. Because source trustworthiness was the strongest antecedent of performance expectancy, platforms should clearly display institutional backing, medical review procedures, professional credentials, and quality assurance mechanisms. In AI-driven healthcare, users may be more willing to perceive a service as useful when they know that the information is supported by credible healthcare institutions or professional oversight.

Second, platforms should improve information quality while making the basis of AI-generated responses more transparent. Users need accurate, relevant, and actionable health information. For higher e-Health Literacy users, high-quality information may be especially important because these users are more capable of evaluating and applying such information. Platforms should therefore provide explanations, references to clinical guidelines where appropriate, and clear boundaries regarding what AI-generated advice can and cannot do.

Third, ease of use remains important, particularly for users with lower e-Health Literacy. Developers should reduce unnecessary operational complexity, simplify navigation, provide plain-language explanations, and offer voice-based or assisted interaction options. For older adults and users with limited digital health capability, clear interface design may reduce cognitive burden and support continued use.

Fourth, privacy protection should be made visible and understandable. Because perceived privacy risk negatively influenced continuance intention, platforms should clearly communicate what data are collected, how data are stored, who can access them, and how users can control their information. Privacy policies should be written in accessible language rather than only in legalistic terms. Visible privacy safeguards may help reduce uncertainty and support sustained engagement.

### Limitations and future research

This study has several limitations. First, the cross-sectional design limits causal inference. Although the proposed model is theoretically grounded, longitudinal research is needed to examine how users’ perceptions and continuance intention evolve over time.

Second, the data were self-reported and collected at one time point. Although procedural remedies, Harman’s single-factor test, collinearity diagnostics, nonresponse bias testing, and data-collection mode comparison were conducted, common method bias cannot be completely ruled out. Future studies could combine survey data with behavioral usage logs, platform usage records, or experimental designs to provide stronger evidence.

Third, although this study was motivated by literacy-related differences in AI-driven healthcare use, it operationalized this issue primarily through e-Health Literacy rather than through a comprehensive set of structural digital divide indicators such as income, rural–urban residence, device access, or social support. Therefore, the findings should be interpreted as evidence of e-Health-Literacy-related heterogeneity rather than as a complete assessment of the digital divide. Future studies should combine literacy measures with subgroup analyses based on age, socioeconomic status, chronic disease status, and access to digital devices.

Fourth, the measurement of perceived interactivity focused mainly on responsiveness, real-time feedback, and dynamic interaction. It did not fully capture all dimensions of interactivity, such as user control and bidirectional communication. Future research should develop a more comprehensive operationalization of interactivity in AI-driven healthcare contexts.

Fifth, the sample was collected in Shanghai, a highly developed urban setting with relatively advanced digital health infrastructure. The findings may not be directly generalizable to rural regions, less-developed areas, or healthcare systems with different digital infrastructure. Future studies should compare users across different regions, service platforms, and healthcare systems.

## Conclusion

This study examined continuance intention toward AI-driven healthcare services among 580 users in Shanghai, China. By integrating UTAUT, IAM, and Privacy Calculus Theory, the study shows that users’ continuance intention is shaped by perceived usefulness, ease of use, perceived interactivity, and privacy risk. Source trustworthiness emerged as the strongest antecedent of performance expectancy, suggesting the importance of credible institutional backing in AI-mediated health contexts.

The study further found that e-Health Literacy modestly but significantly moderated the effects of information quality and effort expectancy. Specifically, the relationship between information quality and performance expectancy was stronger among users with higher e-Health Literacy, whereas the relationship between effort expectancy and continuance intention was stronger among users with lower e-Health Literacy. These findings suggest that users with different levels of health-related digital capability may evaluate AI-driven healthcare services differently.

Overall, this study contributes to a more nuanced understanding of post-adoption behavior in AI-driven healthcare. The findings suggest that inclusive service design should combine trustworthy information, privacy protection, and usability support for users with lower e-Health Literacy. At the same time, the small effect sizes of the moderation effects indicate that the findings should be interpreted cautiously and further validated in future longitudinal and multi-context studies.

## Data Availability

The datasets generated and/or analyzed during the current study are not publicly available due to participant privacy but are available from the corresponding author on reasonable request.

## Appendix A. Survey Instrument

**Table:**

Construct	Code	Item Statement
Source Trustworthiness	ST1	I trust the health content that is provided by the digital health platform.
	ST2	The information I receive from this platform is dependable and authoritative.
	ST3	I believe the medical institution backing this platform is trustworthy.
Information Quality	IQ1	The health advice provided by the platform is accurate and correct.
	IQ2	The information provided by the platform is relevant to my health needs.
	IQ3	The diagnosis or triage suggestions provided by the AI are reliable.
Perceived Interactivity	PI1	The AI assistant on the platform responds to my queries in real-time.
	PI2	The platform understands my specific health questions accurately.
	PI3	I feel that the interaction with the digital health system is dynamic and responsive.
Performance Expectancy	PE1	Using the digital health platform helps me manage my health more effectively.
	PE2	The platform allows me to accomplish healthcare tasks (e.g., booking, inquiry) more quickly.
	PE3	Overall, the platform is useful for my daily health management.
Effort Expectancy	EE1	Learning how to use the digital health platform is easy for me.
	EE2	The interface of the platform is clear and understandable.
	EE3	It is easy for me to become skillful at using the platform without assistance.
Perceived Privacy Risk	PPR1	I am concerned that the digital health platform collects too much personal information.
	PPR2	I worry about the potential loss of privacy when using this platform.
	PPR3	I feel insecure about sending my sensitive health data over this system.
e-Health Literacy	eHL1	I know how to find helpful health resources on the internet.
	eHL2	I have the skills to evaluate the health resources I find on the digital platform.
	eHL3	I feel confident in using information from the internet to make health decisions.
Continuance Intention	CI1	I intend to continue using the digital health platform in the future.
	CI2	I will frequently use this platform for health consultations or appointments.
	CI3	I would recommend this digital health platform to others.
